# Global Drivers of Plant–Pollinator Interaction Specialization in Gardens

**DOI:** 10.1002/ece3.73341

**Published:** 2026-04-06

**Authors:** Luis Gustavo de Sousa Perugini, André Rodrigo Rech, Jeff. Ollerton, Leonardo Ré Jorge

**Affiliations:** ^1^ Programa de Pós‐Graduação em Ciência Florestal Universidade Federal dos Vales do Jequitinhonha e Mucuri Diamantina Brazil; ^2^ Institute of Entomology, Biology Centre, Czech Academy of Sciences České Budějovice Czech Republic; ^3^ Department of Zoology, Faculty of Sciences University of South Bohemia České Budějovice Czech Republic; ^4^ Faculty of Arts, Science and Technology The University of Northampton Northampton UK; ^5^ Kunming Institute of Botany Kunming China

**Keywords:** ecological specialization, interaction network, precipitation, species richness, urban–rural gradient

## Abstract

The frequency of plant–pollinator interactions is shaped by abiotic (e.g., climate and land use characteristics) and biotic factors (e.g., morphological traits, evolutionary history). When applied to gardens size, the degree of urbanization and climate likely influence species richness and interaction specialization. We hypothesize that specialization will be higher in gardens with high species richness and warmer, wetter climates. Additionally, phylogenetically related plants would show similar specialization levels. We further predict that both plant and pollinator richness increase in larger and less urbanized sites. To test these predictions, we analyzed a global dataset of plant–pollinator interactions sampled in garden environments. We considered garden characteristics such as size and type (urban, suburban, rural), and annual mean temperature and precipitation within a causal framework. Additionally, we examined how species richness and phylogenetic relatedness influenced plant specialization (*d*'). Our analysis of 40 plant–pollinator networks revealed that plant species richness was significantly influenced only by garden size and the degree of urbanization, with larger gardens supporting higher richness, and suburban gardens hosting more plant species than both rural and highly urbanized ones. Plant richness and precipitation positively influenced pollinator richness, but no association was found between specialization and environmental variables. Furthermore, the high species‐specific variation in specialization with no phylogenetic signal implies that other plant traits than phylogeny could be driving plant‐pollinator specialization in these systems. Our results suggest distinct factors drive species diversity and interaction specialization in urban gardens.

## Graphical abstract

Using global citizen science data from the Covid lockdown, we show that garden plant and pollinator richness respond to distinct ecological and human factors, while specialization is species‐specific.

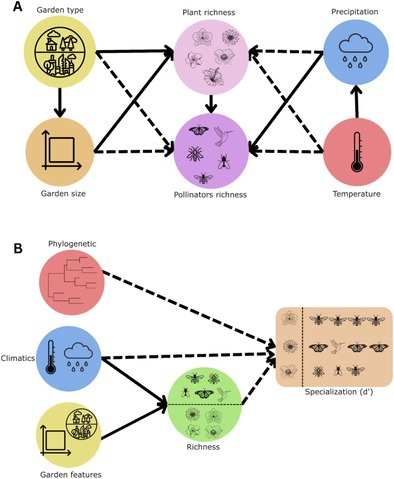

## 1 Introduction

Plant–pollinator interactions may vary according to species richness and composition (Morrison and Mendenhall 2020; Alue et al. 2022; Sánchez‐Dávila et al. 2024). Gardens are unique modified microenvironments that allow the study of plant–animal interactions in a constructed plant community. These communities can vary widely in complexity, garden features, and management practices, which are expected to influence flower visitor behavior and, consequently, the frequency and specialization of plant–pollinator interactions (Baldock et al. 2019; Schmack and Egerer 2023).

The specialization of interactions in gardens can be influenced by flower richness, as pollinators may change their diet based on available resources (Gómez‐Martínez et al. 2022). Increased floral richness and abundance strongly enhance flower visitor interactions, regardless of the surrounding landscape context (Plant et al. 2025). Therefore, garden management practices linked to introducing or removing species change interaction patterns (Seitz et al. 2020), including specialization (Martins et al. 2017). Plant‐pollinator network structure can vary according to features of the garden and the landscape it is located in. The garden area stands out among these drivers, as species‐area relationships are widespread in ecological systems, and also in gardens (Smith et al. 2006; Matteson and Langellotto 2010; Padullés Cubino et al. 2019). The intensity of this pattern may also be influenced by management practices of the garden and its surroundings (Sierra‐Guerrero and Amarillo‐Suárez 2017; Egerer et al. 2019).

With regard to the effect of surrounding landscape on interactions in gardens, rural sites usually harbor greater flower diversity than urban and suburban areas, as building density increases and limits the space available for plant establishment (Graves et al. 2017; Birdshire et al. 2020). On the other hand, urban areas sometimes can host great plant diversity due to an increase in the number of non‐native species added by gardeners (Baldock et al. 2015). Hence, areas with intense land use and lower flower diversity were already reported to host depauperate pollinator faunas (Ganuza et al. 2022). However, this pattern is not universal. Some urban areas, especially those with diverse and well‐managed green spaces, may support a high richness and abundance of certain pollinator taxa (Baldock et al. 2015). These contrasting findings highlight that the effects of land use on pollinator communities may be context‐dependent (Bates et al. 2011; Theodorou et al. 2020; Birdshire et al. 2020) and that local features and the type of landscape need to be assessed independently.

Climate is a crucial factor determining plant and pollinator communities worldwide (Rech et al. 2016). Following common macroecological patterns, pollinator and plant richness generally increase with higher temperature and precipitation (Hawkins et al. 2003; Brown 2014). Some groups, however, show richness peaks in other conditions; for example, bee richness may be higher in warm, temperate, and arid regions (Michener 2007; Trøjelsgaard and Olesen 2013; Orr et al. 2021). Moreover, plant survival in gardens may be affected by several factors, including low‐temperature tolerance (Niinemets and Peñuelas 2008; Padullés Cubino et al. 2019). In this context, seasonality is also supposed to intensify the variations in plant–pollinator interactions within gardens over time.

Specialization quantifies how strongly species interaction partners are used in proportion to their availability (Jorge et al. 2014). Plant specialization tends to increase with pollinator richness and network size, as the relative proportion of partners used by each species decreases, resulting in higher specialization (Xiao et al. 2017). Additionally, in an abundant and highly variable pollinator community, plants require strategies to filter pollinators and minimize the interference of heterospecific pollen (Xiao et al. 2017). Thus, tropical regions, with their higher species richness, are expected to be more specialized, with plants having fewer interaction partners. Previous studies have shown latitudinal patterns in plant‐pollinator specialization to be driven by precipitation (Dalsgaard et al. 2011; Trøjelsgaard and Olesen 2013) and historic climate change velocity (Dalsgaard et al. 2011). However, other studies have shown conflicting patterns (Moles and Ollerton 2016; Xiao et al. 2017; Gorostiague et al. 2023), depending on the plants that are studied and the assumptions made within analyses. It is also unclear if and how these patterns manifest in garden environments.

Plant traits can have different levels of phylogenetic signal (Ortiz et al. 2021). When phylogenetic conservatism occurs, closely related plants share similar characteristics, but morphological convergence may also produce similar traits in unrelated lineages (Junker et al. 2015). Additionally, pollinators have morphological and behavioral constraints that will force them to visit flowers with particular morphologies. Thus, pollinators will show specialization towards phylogenetically related species only if the selective traits show strong phylogenetic signal (Vamosi et al. 2014; Maglianesi et al. 2024). In gardens, the pool of plant species available for a given pollinator is not a result of ecological processes, as they are decided by gardeners based on other criteria, for example, flower size and color (Kendal et al. 2012; Schueller et al. 2023). This will break natural relatedness patterns of species in the community, but pollinators will still be attracted to plants that they are able to visit successfully. Pollinators establish their preferences and foraging intensity on the available resources, and this process will generate the specialization patterns within the constraints established by the plant community selected by gardeners.

Our study aims to understand whether the richness of pollinators and plants in gardens and plant interaction specialization could be driven by environmental variables, garden properties (size, urbanization level), and plant species relatedness. We analyzed the role of abiotic factors (precipitation, temperature, urbanization level, garden size of the property) and plant relatedness on species richness and plant ecological specialization measured from observed interactions. We hypothesized that gardens would have higher species richness when larger and located in less urbanized landscapes (Figure S1A). Even though there is conflicting evidence in the literature, we also hypothesized that specialization would be higher in gardens from warmer and wetter areas, with higher species richness (Figure S1B).

## 2 Material and Methods

### 2.1 Plant–Pollinator Interaction Data

We used the global garden plant–pollinator interaction dataset collected by Ollerton et al. (2022). This study included three types of sampling design, but we focus here on type A. In this design, observers walked slowly around a garden for a fixed period of time, recording every potential pollinator interacting with particular flowers. Survey duration was proportional to garden size and the number of flowering plants, ensuring that sampling effort scaled with habitat area (Ollerton et al. 2022). The dataset is made from observations between March and October 2020, and we excluded gardens where the number of plant or pollinator species was < 5 to avoid undersampled gardens where interaction structure estimation would be biased. Pollinators and plants were identified to the lowest possible taxonomic level according to observer expertise. For plants, a total of 653 morphospecies were recorded; 530 (81%) were identified at the species level, 122 (18%) at the genus level, and only 1 (0.15%) at the family level. On the other hand, among the 661 pollinator morphospecies, 313 (47%) were identified at the species level, 298 (45%) at the genus level, and the remaining 50 (7%) were classified as morphotypes of higher taxonomic level. This shows that plants were identified at a much higher taxonomic resolution than pollinators.

**FIGURE 1:**
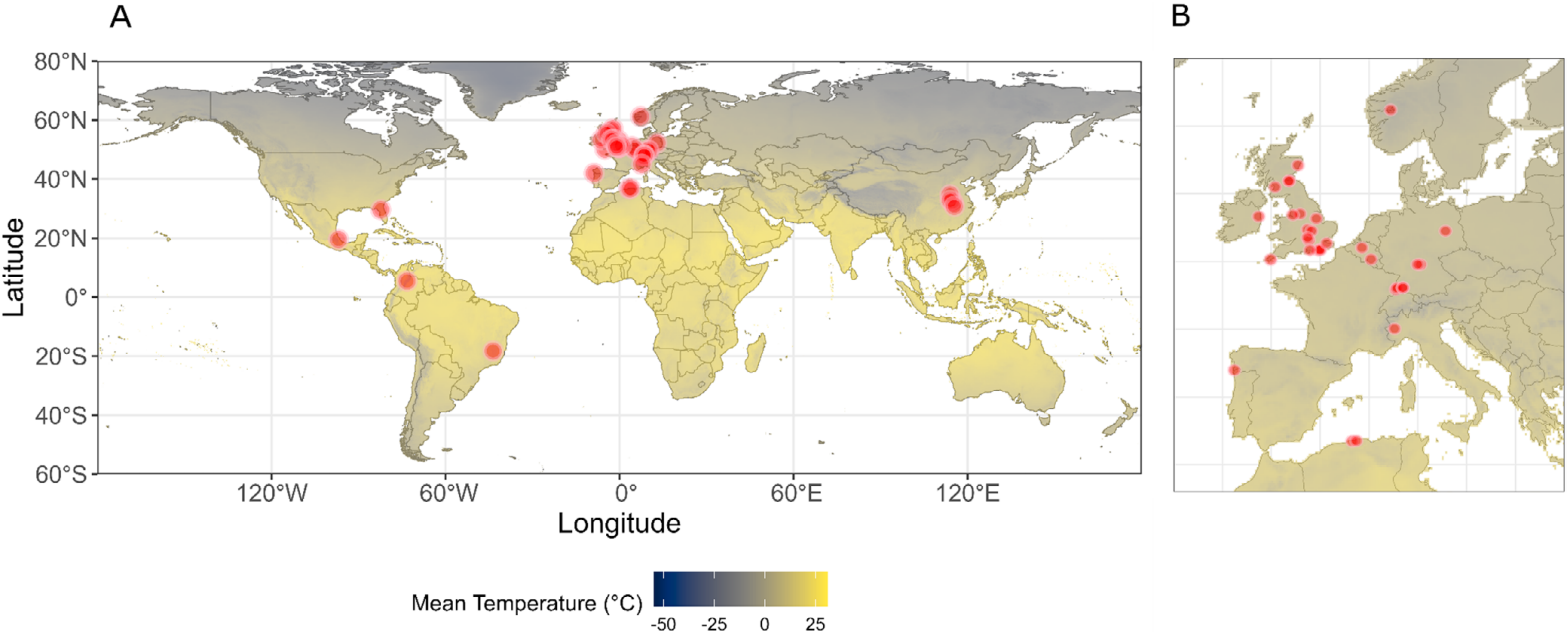
Geographical distribution of the networks analyzed in this study, sourced from the Ollerton et al. (2022) dataset. (A) Global map showing the locations of sampled networks (red dots) collected by researchers between March and October 2020. Local mean annual temperatures are overlaid based on WorldClim data. (B) Focused view of the European region, highlighting the network sampling sites in greater detail.

When assessing plant richness in each garden, we also considered species that were flowering but did not receive any visits by pollinators. The final dataset contained 40 gardens on four continents, with the majority (30) in Europe. From the remaining locations, four were in China, two in Algeria, and one each in Brazil, Colombia, Mexico and the United States (Figure 1). All sites were sampled following the same standardized protocol. All regions with marked seasonality were in the northern hemisphere, ensuring comparability across sites.

### 2.2 Environmental Variables

Garden descriptors are summarized in Table S1 and were provided by each garden owner. Garden size was defined as the total garden area (m^2^), while garden type was defined according to the degree of urbanization surrounding each garden, as reported by their owners, and categorized as rural, suburban, or urban. Bioclimatic layers were obtained from the WorldClim database based on each garden's geographic coordinates (longitude and latitude). The bioclimatic variables selected from WorldClim included annual mean temperature and annual precipitation. Species richness of pollinators and plants were defined as the total number of species recorded in each network.

To assess species‐level differences in diet specialization, we built a quantitative bipartite interaction matrix for each of the 40 gardens, with the total number of visits recorded as the weight of each interaction. Using the R package bipartite, we chose the *d*' reciprocal specialization index (Blüthgen et al. 2006) to measure specialization for all plants in each garden (Dormann et al. 2026). This index varies between 0 and 1, where zero means maximum generalization and one is a pair of species that interact mutually exclusively (Blüthgen et al. 2006). We focused on plants due to the greater clarity and consistency in species identification and delimitation across sites. The morpho‐speciation of the pollinators, on the other hand, is more difficult, and the small proportion of and inconsistencies between different sites hindered comparing the same pollinators across networks.

### 2.3 Data Analysis

To investigate the relationship between garden plant richness and their potential drivers (Figure S1A), we used generalized linear models with a negative binomial distribution with the following predictors: mean annual temperature, mean annual precipitation, type of garden (rural, suburban, and urban), the logarithm of garden area, and the interaction between garden type and size. To assess which factors were relevant, we used model selection, comparing all possible variable combinations from this full model using package *MuMIn*. With the best performing model, we tested for pairwise differences between garden types using package *emmeans* (Lenth 2023) and Tukey correction for multiple comparison estimates. We used the same approach as above to model pollinator richness, changing only the predictor variables by adding plant richness along with the ones used in the previous model.

Additionally, aiming to assess the drivers of plant specialization (Figure S1B), within (the same species occurring in different sites) and between plant species across all networks, we used Bayesian regression models with the package *brms*. We started by assessing whether there was a phylogenetic signal in specialization, or if most of its variation had a non‐phylogenetic (or intraspecific) origin. For that, we first built a plant phylogeny using the *V.phyloMaker* package (Jin and Qian 2022), based on the species lists of all networks. This uses the supertree compiled by Jin and Qian (2022) to build a tree with all species in the study, including branch lengths. From this phylogeny, we obtained a variance–covariance matrix representing the phylogenetic correlations among species. We then built a mixed effects model, with *d*' as the response variable, with a beta distribution, and only two random effects as predictors: species as a categorical variable and species accounting for their correlation using the phylogenetic variance–covariance matrix. As we observed a negligible effect of the phylogenetic component, indicating no detectable phylogenetic structure in specialization, we followed further analysis only with species as a simple random effect. We then built the main model for the analysis of specialization. This model also used *d*' with a beta distribution as the response variable and included the following fixed predictors: the logarithm of the combined plant and pollinator species richness, annual mean precipitation, and annual mean temperature, as well as the interactions between species richness and annual precipitation, and between species richness and annual temperature. As random effects, we used species and network. We fitted all models using eight chains, each with 1250 iterations, following a warmup of 1000 iterations. Diagnostic plots were generated to ensure model convergence and adequacy of fit (Figures S2 and S3). All the analyses were conducted in the R environment (R Development Core Team 2022).

## 3 Results

### 3.1 Species Richness

The data set included 4302 interactions across 40 networks, involving 653 plant and 661 pollinator species. The number of interactions per garden ranged from 10 to 373, with 16 gardens having fewer than 50 interactions. Pollinator richness varied from 8 to 179 species per garden, while plant richness ranged from 6 to 125 species. On average, each plant species was visited by 4 pollinator species, while each pollinator species visited 2.99 plant species. Individuals of *Taraxacum officinale* F.H. Wigg. (Asteraceae), the most frequent plant species, were present in 15 gardens, with 164 interactions observed. *Apis mellifera* L. (Hymenoptera: Apidae) was the most frequent pollinator, with 337 interactions and appearing in 35 different gardens.

**FIGURE 2:**
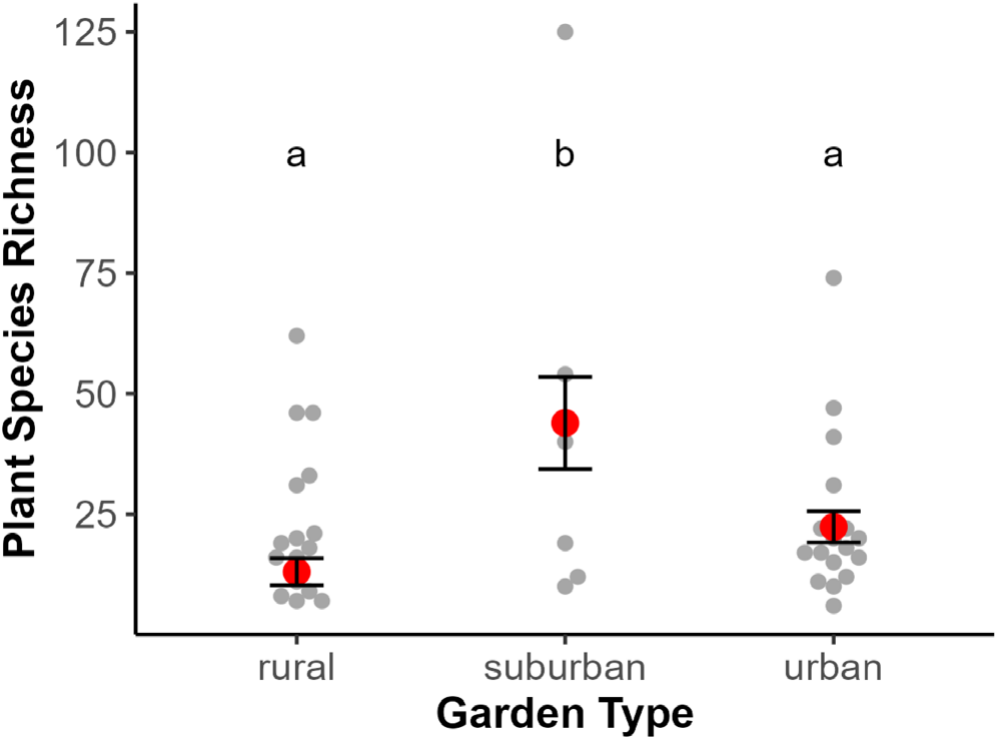
Variation in plant species richness across urbanization level of 40 gardens worldwide. Each gray point represents the plant species richness observed in an individual garden. Red points indicate the mean plant richness estimated from a linear urbanization‐level model, with error bars representing the 95% confidence intervals around the mean. Different letters indicate statistically significant differences between garden types based on a multiple comparisons test.

**FIGURE 3:**
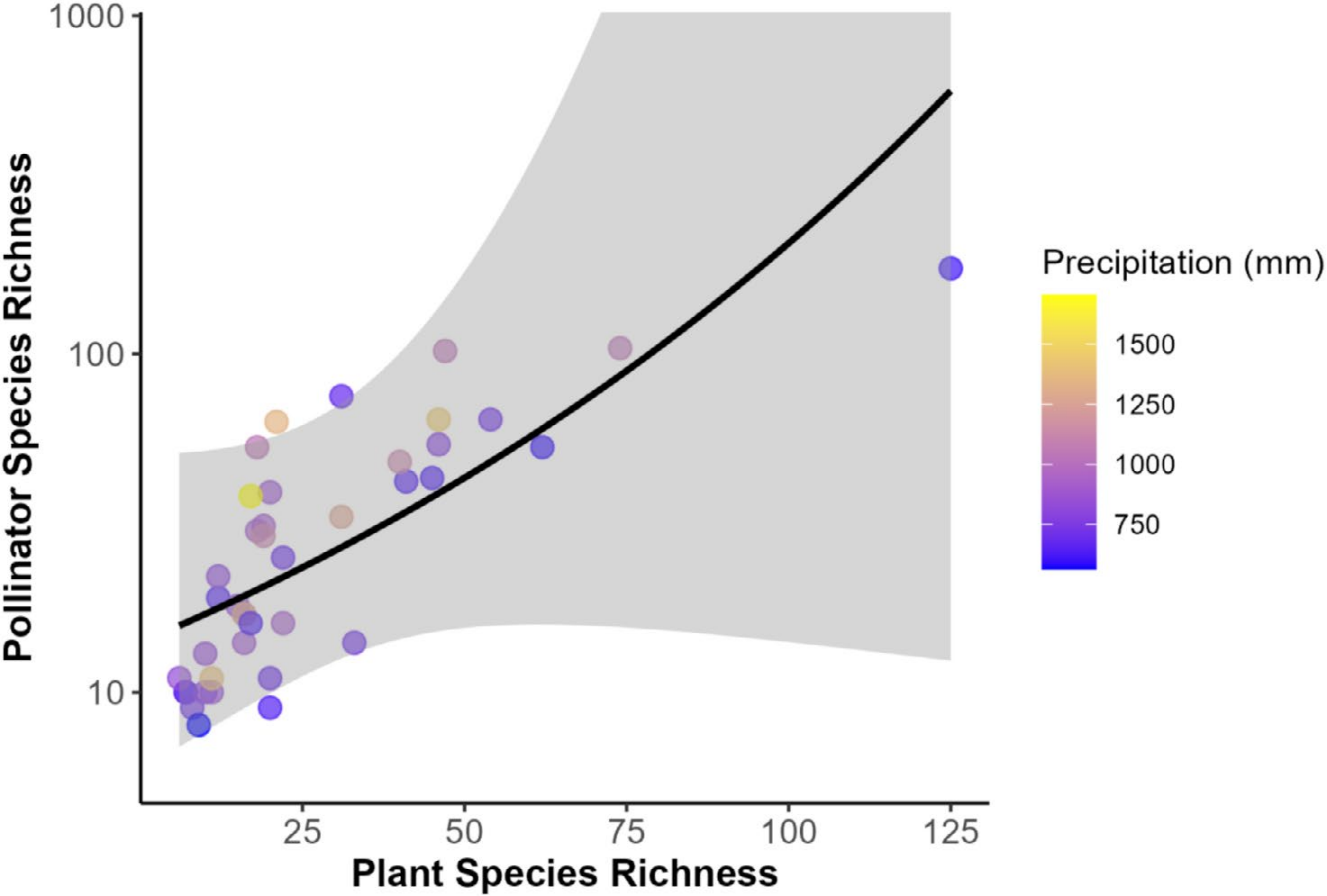
Relationship between plant and pollinator species richness across different precipitation levels. Each point represents a garden, with plant species richness on the x‐axis and pollinator species richness (log‐transformed) on the y‐axis. Precipitation values represent annual totals (mm) and are indicated by the color gradient, with yellow representing higher precipitation and blue having lower precipitation. The black line represents the linear regression model, with the shaded area indicating the 95% confidence interval.

Our analysis revealed that climate does not affect the richness of plant species. However, there was a relationship between urbanization level and plant species richness, with suburban gardens richer in species (43.9 ± 9.56, mean ± SD) than rural (13.0 ± 2.79, *p* = 0.002) and urban (22.4 ± 3.23, *p* < 0.03) (Figure 2 and Tables S2 and S3). Additionally, we found a positive influence of garden size in plant richness (*p* < 0.003 ± 0.12). Given size and urbanization were added to the same model, their effects on plant richness are independent and direct. For pollinator richness, we found it to be positively affected by plant richness and precipitation (Figure 3 and Table S4). This indicates that gardens with a greater variety of plant species and those in areas with higher precipitation support a more diverse pollinator community.

### 3.2 Specialization

Across all networks, 58 plants showed maximum generalization (*d*' = 1), while 59 plants had minimum specialization (*d*' = 0). On average, plants interacted with 10.16% of the pollinator community, with a mean *d*' of 0.39 ± 0.26. Additionally, 22 gardens had the full range of specialization for their plant species, while five had at least one species with maximum specialization, and six had species with minimum specialization. In the phylogenetic mixed‐effects model, we observed a negligible phylogenetic signal in specialization, with most of the variability captured by the non‐phylogenetic component (Table S5A), suggesting that differences between species are mostly idiosyncratic. This allowed us to follow up with further analyses using a simpler model that disregarded the phylogenetic correlation between species.

When including all predictors of plant specialization in the mixed‐effects model, we observed considerable variability both between networks and between species (Table S5B). However, none of the fixed effects considered here showed clear effects on specialization, with all confidence intervals including zero. This suggests that neither species richness nor environmental variables significantly explain specialization in these gardens, with the variability in specialization driven by particular species and garden properties.

## 4 Discussion

By using a globally standardized dataset of urban gardens, this study avoids the inconsistencies often associated with literature‐based comparisons and provides more robust and comparable results. We found no apparent effect of climate on garden plant richness, but larger and suburban gardens have more plant species than smaller and rural or urban gardens. However, plant richness was the main driver for pollinator richness, with a smaller effect of local precipitation. Plant specialization was unrelated to climate variables or community species richness and showed no phylogenetic signal. Therefore, environmental factors as precipitation and garden size affecting species richness in gardens do not directly impact plant specialization in this particular environment, suggesting a mismatch between the processes driving species diversity and those shaping interaction specialization in gardens.

### 4.1 Plant Richness

We found that suburban gardens are richer in plant species than rural and urban gardens. A likely explanation for this finding is that rural gardens are more isolated, with a lower frequency of species dispersion between gardens, while in suburban and urban areas, there can be multiple gardens within close distance, allowing dispersion (von der Lippe and Kowarik 2008; Knapp et al. 2012). Conversely, while urban gardens also share this higher dispersion, they suffer more from the negative effects of urbanization and garden size (Lin et al. 2018). Moreover, urban and rural areas may differ in their goals for the garden according to the owner's preferences (Lynch et al. 2021).

The positive relationship between garden size and species richness reflects a pattern widely documented in urban contexts around the world (Bernholt et al. 2009; Huai et al. 2011). Larger gardens provide more physical space for the establishment and coexistence of multiple plant types, while also helping to preserve natural vegetation by avoiding disturbances associated with its removal (Patel et al. 2022). Additionally, larger gardens support a wider range of land cover types (tall trees, vegetable patches, compost heaps), which are key features that increase habitat heterogeneity and resource availability for both cultivated and spontaneous plant species (Smith et al. 2006).

Our findings show that climate does not influence plant richness in gardens. Given that gardens are artificial communities, natural filtering effects caused by the climate are buffered by the fact that plants are selected based on human preferences (Kendal et al. 2012). Regardless of climate, human preference for specific traits and management habits, such as the time spent gardening or if the plants are functional for humans (e.g., shade provision or fruits), should influence the species selected to compose gardens and consequently, plant species richness in the site (Kendal et al. 2012; Egerer et al. 2019; Philpott et al. 2020; Neumann et al. 2024). The temperature in gardens can be influenced by local factors, such as the size of the garden and the presence of trees, grass, and rocks (Lin et al. 2018). Temperature variability can change the behavior of gardeners due to the differential need for watering or plant species selection, which can influence the richness of the species within gardens (Egerer et al. 2019). Moreover, higher imperviousness negatively influences plant species richness. In areas with lower levels of impervious surfaces, water can penetrate the soil more easily, allowing plants to access diverse habitats promoting different ecological niches (Seitz et al. 2020). This implies that even in regions with high precipitation if impermeable surfaces dominate the environment, the richness of plant species may still be reduced due to limited water infiltration and habitat availability.

### 4.2 Pollinator Richness

We found a positive influence of plant richness and precipitation on pollinator richness. Plant richness commonly positively influences pollinators' richness within gardens, as more plant species can attract a wider variety of pollinators (Pardee and Philpott 2014; Watson et al. 2022). This influence of plants on pollinator richness may be explained by the high diversity of plants extending and diversifying the resource availability used by pollinators over time (Majewska and Altizer 2020; Stewart and Waitayachart 2020; Tew et al. 2022). Moreover, it is not just related to food resources but also to providing shade, protection from winds, and nest sites (Majewska and Altizer 2020). In agreement with other studies, garden size was unrelated to visitor diversity (Tasker et al. 2020). Other factors, such as the heterogeneity of the vegetation (as likely reflected in plant richness), have already been shown to be more influential in pollinator diversity, regardless of the garden size (Gunnarsson and Federsel 2014). Other local factors could influence pollinators in gardens, such as the origin of the flowers, as native and exotic plants can differ in their influence on the availability of resources during the seasons for pollinators (Pardee and Philpott 2014; Staab et al. 2020) or floral resource abundance (Plascencia and Philpott 2017).

Finally, we found greater pollinator diversity in places with higher precipitation. As nutrients and water limit angiosperms, precipitation may drive primary investments of plants in reproductive structures and strategies (Rech et al. 2016), such as floral abundance, corolla length and width, and nectar volume and concentration (Gallagher and Campbell 2017; Phillips et al. 2018). Thus, the higher investments of the plant in sexual reproduction will positively influence pollinator richness. One way precipitation can influence pollinator richness is through nectar; more precipitation may increase nectar volume, influencing pollinator abundance and species richness (Biella et al. 2022). Moreover, the quantity of water in the ground should influence blooming patterns, as drought reduces floral abundance (Phillips et al. 2018) and pollinator communities (Lowenstein et al. 2014). Additionally, pollinator biology can also be influenced by precipitation; nesting behavior, for instance, can be impacted by soil moisture, which is crucial for some species (Dai et al. 2022).

### 4.3 Specialization

Our results show that plant specialization on pollinators in gardens is independent of environmental variables or community context, suggesting it is primarily driven by species‐specific traits such as morphology and phenology (Galetto et al. 2023). Interestingly, local species richness does not seem to influence garden plant specialization. However, other factors related to garden composition and management practices, such as variations in floral abundance due to mowing, can affect the resources available and, consequently, the interactions between plants and pollinators (Lerman et al. 2018; Lynch et al. 2021). Additionally, surrounding flowers can impact the likelihood and frequency of visits that a particular plant receives (Fowler et al. 2016; Berthon et al. 2021). These findings suggest a distinction between the factors driving species diversity and those shaping plant interaction specialization in gardens.

Even with the specialization not defined by a latitudinal pattern (Ollerton et al. 2022), environmental variables that change with latitude may influence specialization and deserve attention under climate change, potentially disrupting stable interactions in climatically stable regions (Dalsgaard et al. 2011). Environmental variables showed neither a direct nor an indirect effect on specialization in plant‐pollinator interactions. In gardens, environmental conditions can be manipulated with regular irrigation and protection against extreme climatic variations, which reduces the direct influence of climatic factors such as temperature and precipitation (Lin et al. 2018). Plant specialization showed no phylogenetic signal, while it was strongly species‐specific. This suggests that the species traits driving specialization also have low phylogenetic signals, as observed in other studies (e.g., Maglianesi et al. 2024). Therefore, when considering specialization, it is essential to consider various non‐phylogenetic factors, including specific plant characteristics like floral traits and phenology not necessarily correlated to phylogenetic relatedness (Ortiz et al. 2021). Additionally, phylogeny might have especially low importance for garden cultivars and hybrids, as these often have very different traits compared to their wild ancestors, further breaking the phylogenetic signal of these traits (Mitchell et al. 2019).

### 4.4 Study Limitations and Perspectives

Even though this study was conducted in 40 locations across a wide range of latitudes and biogeographic regions, the sampling effort is concentrated in Europe and the Northern Hemisphere. This bias reproduces a common lack of studies in the global South across the literature (Nascimento et al. 2020; Silva et al. 2021). Expanding this protocol to gardens across other regions, mostly in the Southern Hemisphere is an important direction for future research, which could help clarify interaction patterns that are not captured in the currently geographically biased garden networks.

Garden type was used as a coarse proxy for landscape context, as we did not explicitly quantify landscape connectivity or isolation around each site. This limits our ability to directly assess how spatial configuration influences species richness. Future studies should incorporate explicit spatial metrics to more directly test the effects of isolation and landscape context on species richness. Additionally, future studies could incorporate plant traits to evaluate whether species with similar specialization share traits. For instance, it is still unclear whether there are links between their cultivated status and level of specialization. Notwithstanding, it is still unknown whether the role of morphological traits in structuring the interactions is widespread in garden communities (Armbruster and Muchhala 2020; Perugini et al. 2025).

Finally, an important avenue of research is the potential mismatch between plants preferred by gardeners and the ones that attract and maintain pollinator communities. Gardeners select plants for esthetic traits that are not necessarily related to pollinator attraction or resource accessibility (Garbuzov and Ratnieks 2014). In addition, exotic ornamental plants may not always provide equivalent resources for native pollinators (Garbuzov et al. 2017). Such mismatches may be further shaped by socioeconomic context, as variation in garden area and planting decisions across socioeconomic settings can influence floral resource availability and, consequently, interaction network structure (Schell et al. 2020; Gomes et al. 2023).

## 5 Conclusion

In conclusion, our study reveals that distinct factors influence plant and pollinator richness in garden ecosystems: while urbanization levels primarily drive plant richness, pollinator richness is driven by plant richness and precipitation. However, the apparent influence of these factors on species richness does not directly impact plant specialization, which appears to be more associated with species‐specific traits rather than environmental factors in gardens. Our findings highlight the complexity of plant–pollinator interactions in anthropogenic landscapes, where human preferences and management practices may shape ecological processes and patterns.

## Author Contributions

**Luis Gustavo de Sousa Perugini:** conceptualization (equal), data curation (equal), investigation (equal), visualization (equal), writing – original draft (equal), writing – review and editing (equal). **André Rodrigo Rech:** funding acquisition (equal), visualization (equal), writing – review and editing (equal). **Jeff. Ollerton:** data curation (equal), writing – review and editing (equal). **Leonardo Ré Jorge:** conceptualization (equal), formal analysis (equal), methodology (equal), supervision (equal), visualization (equal), writing – review and editing (equal).

## Funding

We thank Fundação de Amparo à Pesquisa do Estado de Minas Gerais (#FAPEMIG APQ‐14512‐22 scholarship to LGSP and research projects #APQ‐00932‐21, #APQ‐03100‐21, #RED‐00039‐23, #APQ‐02806‐22, #APQ‐03364‐21, #APQ‐01151‐22, #APQ‐01822‐21), Coordenação de Aperfeiçoamento de Pessoal de Nível Superior—Brasil (CAPES—Finance Code 001), and Conselho Nacional de Desenvolvimento Científico e Tecnológico (CNPq #311665/2022‐5, #400904/2019‐5, #423939/2021‐1 to A.R.R.). J.O. is grateful to the Yunnan Government (China) for support via a High‐end Foreign Expert Project of the Yunnan Revitalization Talent Support Program. L.R.J. was funded by Czech Science Foundation grant GAČR 22‐17593M.

## Conflicts of Interest

The authors declare no conflicts of interest.

## Supporting information


**Figure S1:** Directed acyclic graph (DAG) illustrating the hypothesized relationships among environmental variables and ecological metrics. (A) Effects of garden features and climatic variables on species richness. Variables include garden type (rural, suburban, urban), garden size, plant species richness, pollinator species richness, precipitation, and temperature. Solid arrows represent significant effects, while dashed arrows indicate non‐significant or weaker effects. (B) Influence of phylogenetic structure, climate, garden features, and species richness on plant–pollinator interaction specialization (*d*'). Richness encompasses both plant and pollinator diversity. Dashed arrows indicate hypothesized but non‐significant pathways.
**Figure S2:** Posterior distributions (left panels) and traceplots of the MCMC chains (right panels) for parameters of the Bayesian beta regression model including random intercepts for species (SP) and a phylogenetic effect (Phylo).
**Figure S3:** Posterior distributions (left panels) and MCMC traceplots (right panels) for parameters of the Bayesian beta regression model relating beta diversity to environmental predictors and species richness. The model includes standardized fixed effects of log‐transformed species richness (log_soma.z), temperature (bio1.z), precipitation (bio12.z), their interactions, and random intercepts for species (SP) and sampling network (NetID).
**Table S1:** Characteristics of the Studied Gardens:
**Table S2:** Model selection table for predictors of plant species richness. Models were ranked using AICc, and only models with AIC < 2 are typically considered equally supported. Predictors include garden features, climatic variables, and their interactions.
**Table S3:** Estimated marginal means of pollinator richness across rural, suburban, and urban sites, with pairwise contrasts and 95% confidence intervals.
**Table S4:** Model selection table for predictors of pollinator species richness. Models were ranked using AICc, and only models with AIC < 2 are typically considered equally supported. Predictors include garden features, climatic variables, and their interactions.
**Table S5:** Results of the beta models. (A) Beta model with a phylogenetic structure, testing the influence of phylogeny on d.beta. The model includes random effects for species and the phylogenetic component. (B) Beta model testing the effects of species richness, mean annual temperature, annual precipitation, and their interactions on d.beta. The model includes random effects for species and network.

## Data Availability

All raw data, processed data, and R scripts used for analysis and figure generation are available at Zenodo: https://doi.org/10.5281/zenodo.14662717.
