# Supplementary material for: Global Drivers of Plant–Pollinator Interaction Specialization in Gardens
Source: Ecol Evol. 2026 Apr 6;16(4):e73341. doi: 10.1002/ece3.73341 (PMC13053170; doi:10.1002/ece3.73341)
Supplement: Supplementary file 1 — Figure S1: Directed acyclic graph (DAG) illustrating the hypothesized relationships among environmental variables and ecological metrics. (A) Effects of garden features and climatic variables on species richness. Variables include garden type (rural, suburban, urban), garden size, plant species richness, pollinator species richness, precipitation, and temperature. Solid arrows represent significant effects, while dashed arrows indicate non‐significant or weaker effects. (B) Influence of phylogenetic structure, climate, garden features, and species richness on plant–pollinator interaction specialization (d'). Richness encompasses both plant and pollinator diversity. Dashed arrows indicate hypothesized but non‐significant pathways. Figure S2: Posterior distributions (left panels) and traceplots of the MCMC chains (right panels) for parameters of the Bayesian beta regression model including random intercepts for species (SP) and a phylogenetic effect (Phylo). Figure S3: Posterior distributions (left panels) and MCMC traceplots (right panels) for parameters of the Bayesian beta regression model relating beta diversity to environmental predictors and species richness. The model includes standardized fixed effects of log‐transformed species richness (log_soma.z), temperature (bio1.z), precipitation (bio12.z), their interactions, and random intercepts for species (SP) and sampling network (NetID). Table S1: Characteristics of the Studied Gardens: Table S2: Model selection table for predictors of plant species richness. Models were ranked using AICc, and only models with AIC < 2 are typically considered equally supported. Predictors include garden features, climatic variables, and their interactions. Table S3: Estimated marginal means of pollinator richness across rural, suburban, and urban sites, with pairwise contrasts and 95% confidence intervals. Table S4: Model selection table for predictors of pollinator species richness. Models were ranked using AICc, and only models [file ECE3-16-e73341-s001.docx]

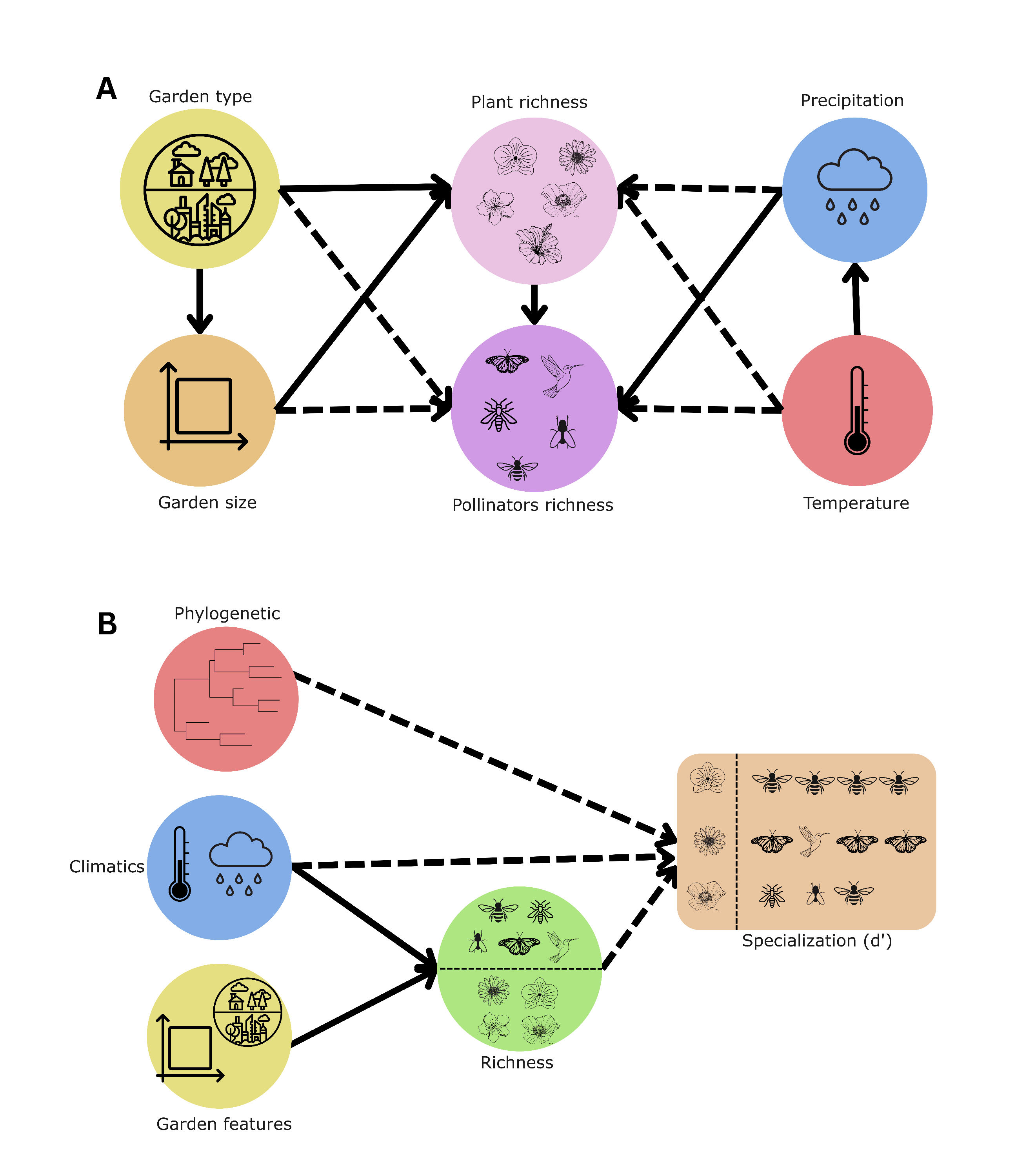


**Figure S1:** Directed acyclic graph (DAG) illustrating the hypothesized relationships among environmental variables and ecological metrics. (A) Effects of garden features and climatic variables on species richness. Variables include garden type (rural, suburban, urban), garden size, plant species richness, pollinator species richness, precipitation, and temperature. Solid arrows represent significant effects, while dashed arrows indicate non-significant or weaker effects. (B) Influence of phylogenetic structure, climate, garden features, and species richness on plant–pollinator interaction specialization (d′). Richness encompasses both plant and pollinator diversity. Dashed arrows indicate hypothesized but non-significant pathways.


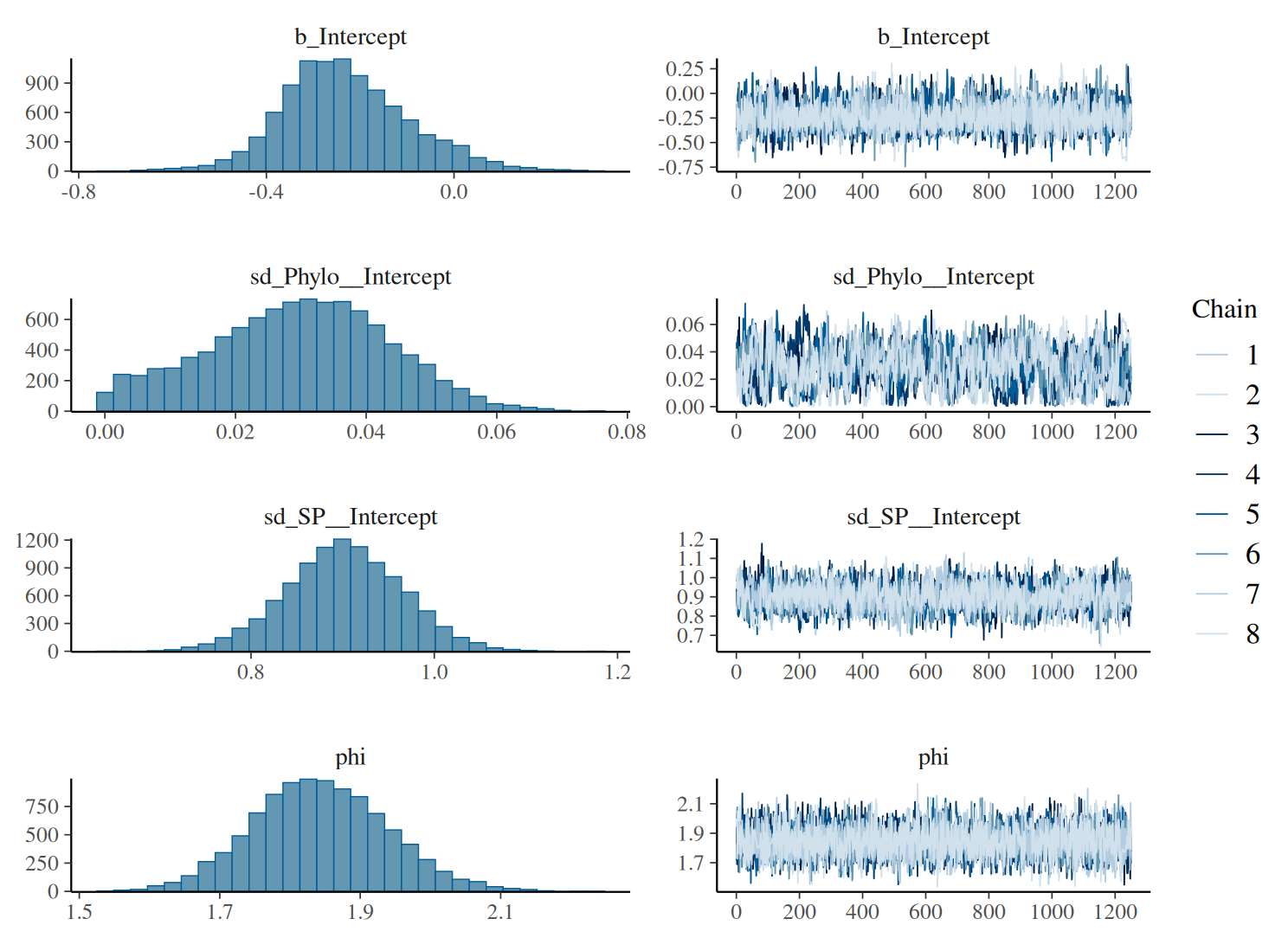


**Figure S2:** Posterior distributions (left panels) and traceplots of the MCMC chains (right panels) for parameters of the Bayesian beta regression model including random intercepts for species (SP) and a phylogenetic effect (Phylo).


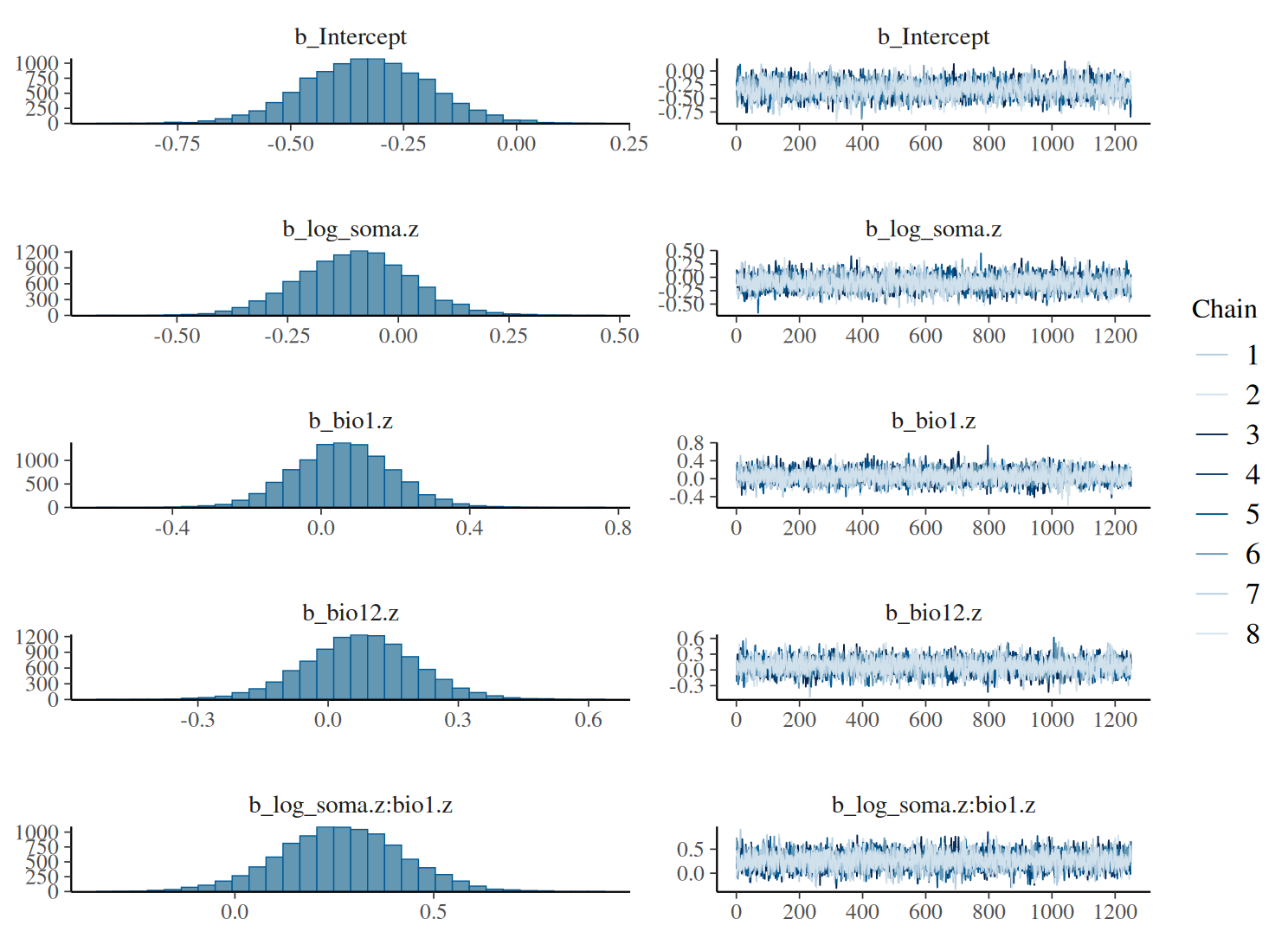

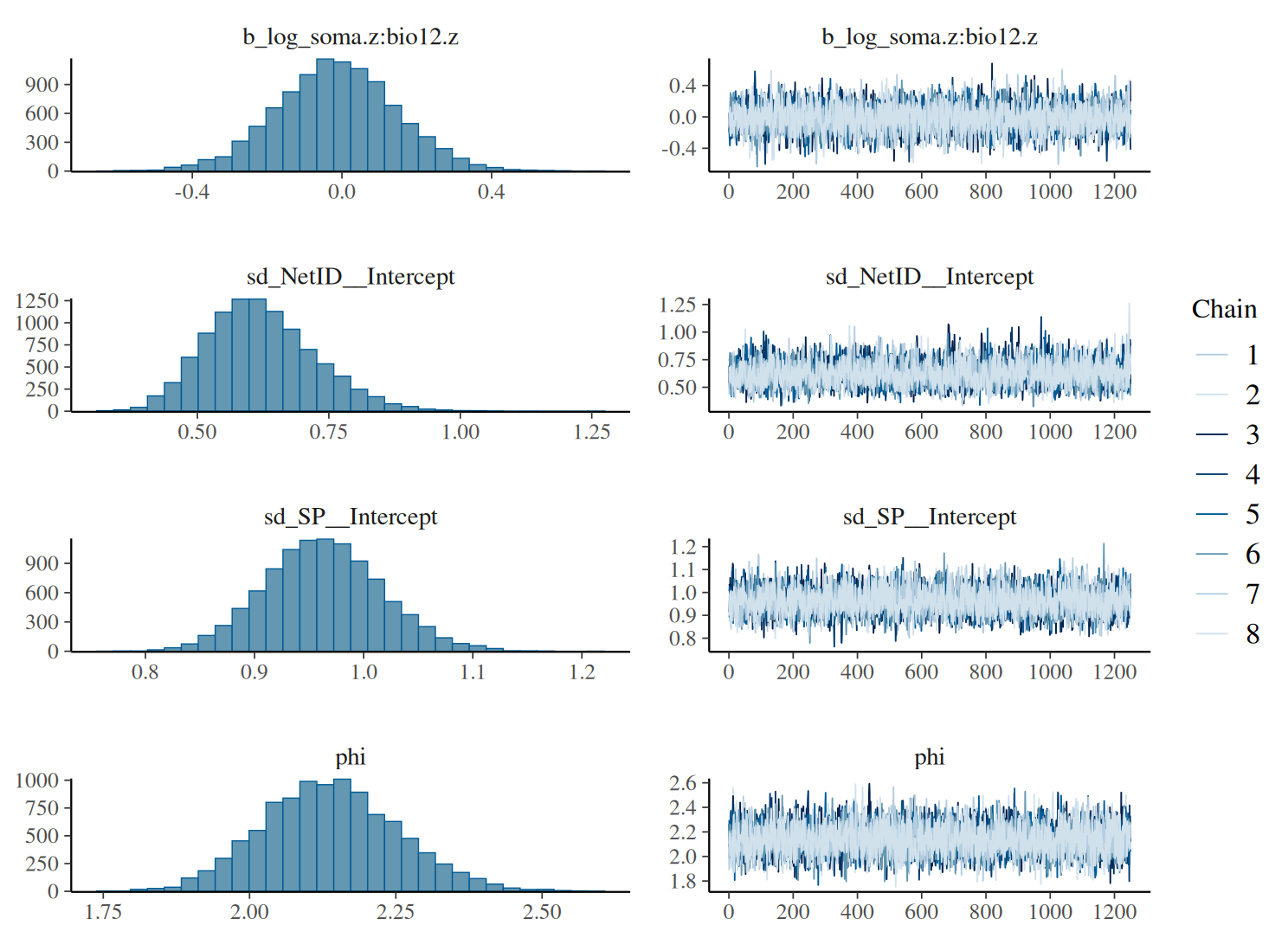
**Figure S3.** Posterior distributions (left panels) and MCMC traceplots (right panels) for parameters of the Bayesian beta regression model relating beta diversity to environmental predictors and species richness. The model includes standardized fixed effects of log-transformed species richness (log_soma.z), temperature (bio1.z), precipitation (bio12.z), their interactions, and random intercepts for species (SP) and sampling network (NetID).

**Table S1:** Characteristics of the Studied Gardens:

| Country | Garden  size (m²) | Type | Animal  Richness | Plant  Richness | Annual mean temperature | Annual mean precipitation |
| --- | --- | --- | --- | --- | --- | --- |
| Algeria | 100 | urban | 75 | 31 | 181 | 672 |
| Algeria | 40 | urban | 18 | 15 | 180 | 832 |
| Belgium | 1422 | rural | 54 | 46 | 103 | 775 |
| Belgium | 400 | rural | 31 | 19 | 92 | 908 |
| Brazil | 8000 | rural | 64 | 46 | 191 | 1447 |
| China | 80 | rural | 17 | 16 | 158 | 1027 |
| China | 1000 | rural | 9 | 20 | 146 | 639 |
| China | 180 | suburban | 22 | 12 | 151 | 806 |
| China | 200 | rural | 11 | 11 | 163 | 1393 |
| Colombia | 130 | rural | 30 | 18 | 129 | 918 |
| Germany | 300 | rural | 10 | 7 | 90 | 612 |
| Germany | 350 | rural | 14 | 16 | 100 | 880 |
| Germany | 120 | urban | 39 | 20 | 102 | 876 |
| Germany | 710 | rural | 8 | 9 | 91 | 563 |
| Germany | 205 | suburban | 43 | 45 | 93 | 621 |
| Germany | 200 | urban | 102 | 47 | 73 | 1047 |
| Germany | 350 | urban | 104 | 74 | 73 | 1047 |
| Ireland | 101.25 | urban | 10 | 11 | 94 | 835 |
| Italy | 370 | urban | 13 | 10 | 126 | 839 |
| Mexico | 435 | urban | 38 | 17 | 177 | 1704 |
| Norway | 1200 | rural | 33 | 31 | 40 | 1270 |
| Spain | 2860 | rural | 63 | 21 | 144 | 1341 |
| UK | 80 | urban | 19 | 12 | 94 | 631 |
| UK | 25 | urban | 53 | 18 | 102 | 1055 |
| UK | 90 | suburban | 64 | 54 | 97 | 699 |
| UK | 250 | rural | 10 | 7 | 98 | 684 |
| UK | 1088 | rural | 14 | 33 | 97 | 716 |
| UK | 84 | rural | 9 | 8 | 97 | 716 |
| UK | 72 | urban | 11 | 20 | 87 | 693 |
| UK | 1000 | rural | 53 | 62 | 96 | 584 |
| UK | 96 | urban | 25 | 22 | 87 | 693 |
| UK | 32 | suburban | 10 | 10 | 78 | 776 |
| UK | 200 | urban | 42 | 41 | 95 | 628 |
| UK | 140 | suburban | 48 | 40 | 82 | 1122 |
| UK | 65 | suburban | 29 | 19 | 90 | 1114 |
| UK | 400 | urban | 16 | 22 | 92 | 915 |
| UK | 80 | suburban | 179 | 125 | 101 | 607 |
| UK | 75 | urban | 16 | 17 | 98 | 619 |
| UK | 142 | urban | 11 | 6 | 102 | 833 |
| USA | 5.161 | urban | 17 | 16 | 204 | 1319 |

**Table S2.** Model selection table for predictors of plant species richness. Models were ranked using AICc, and only models with AIC < 2 are typically considered equally supported. Predictors include garden features, climatic variables, and their interactions.

| **Intercept** | **bio1** | **bio12** | **log Garden.size** | **Type** | **log Garden.size:Type** | **df** | **logLik** | **AICc** | **delta** | **weight** |
| --- | --- | --- | --- | --- | --- | --- | --- | --- | --- | --- |
| 1.248312 |  |  | 0.286489 | + |  | 5 | -159.354 | 330.4724 | 0 | 0.519763 |
| 1.428218 |  | -0.00022 | 0.289782 | + |  | 6 | -159.161 | 332.8673 | 2.39488 | 0.156951 |
| 1.479024 | -0.00132 |  | 0.274103 | + |  | 6 | -159.226 | 332.9968 | 2.524392 | 0.147109 |
| 0.759495 |  |  | 0.362764 | + | + | 7 | -158.971 | 335.4416 | 4.969123 | 0.043329 |
| 1.543434 | -0.00086 | -0.00018 | 0.281281 | + |  | 7 | -159.112 | 335.7241 | 5.25169 | 0.03762 |
| 3.140914 |  |  |  | + |  | 4 | -164.206 | 337.5547 | 7.082252 | 0.015063 |
| 1.026288 | -0.00239 |  | 0.363771 | + | + | 8 | -158.606 | 337.8566 | 7.384128 | 0.012953 |
| 0.930773 |  | -0.00027 | 0.37444 | + | + | 8 | -158.684 | 338.0124 | 7.539945 | 0.011982 |
| 3.290266 |  |  |  |  |  | 2 | -167.035 | 338.3935 | 7.921082 | 0.009903 |
| 3.714978 | -0.00387 |  |  |  |  | 3 | -166.158 | 338.9817 | 8.509305 | 0.00738 |
| 2.746809 |  |  | 0.1007 |  |  | 3 | -166.262 | 339.1912 | 8.718809 | 0.006646 |
| 3.452634 | -0.00266 |  |  | + |  | 5 | -163.732 | 339.2293 | 8.756859 | 0.00652 |
| 3.200316 | -0.00402 |  | 0.098144 |  |  | 4 | -165.301 | 339.7458 | 9.273354 | 0.005036 |
| 3.173198 |  | -3.5E-05 |  | + |  | 5 | -164.202 | 340.1682 | 9.695792 | 0.004077 |
| 3.531535 |  | -0.00028 |  |  |  | 3 | -166.79 | 340.2475 | 9.775111 | 0.003919 |
| 3.024327 |  | -0.00044 | 0.120124 |  |  | 4 | -165.679 | 340.4999 | 10.02743 | 0.003454 |
| 1.089688 | -0.00189 | -0.00019 | 0.372153 | + | + | 9 | -158.473 | 340.9451 | 10.47262 | 0.002765 |
| 3.768243 | -0.00358 | -9.8E-05 |  |  |  | 4 | -166.129 | 341.4016 | 10.92916 | 0.002201 |
| 3.390429 | -0.00292 | 9.94E-05 |  | + |  | 6 | -163.701 | 341.9479 | 11.47548 | 0.001675 |
| 3.283434 | -0.00326 | -0.00027 | 0.110347 |  |  | 5 | -165.104 | 341.9728 | 11.50036 | 0.001654 |

**Table S3.** Estimated marginal means of pollinator richness across rural, suburban, and urban sites, with pairwise contrasts and 95% confidence intervals.

| **Type** | **contrast** | **response** | **SE** | **df** | **asymp.LCL** | **asymp.UCL** |
| --- | --- | --- | --- | --- | --- | --- |
| rural | . | 13.03508 | 2.794356 | Inf | 7.404406 | 22.94758 |
| suburban | . | 43.91506 | 9.556803 | Inf | 24.73253 | 77.97555 |
| urban | . | 22.35762 | 3.233215 | Inf | 15.26617 | 32.74319 |
| . | rural - suburban | 0.296825 | 0.091055 | Inf | 0.132135 | 0.666782 |
| . | rural - urban | 0.583026 | 0.147084 | Inf | 0.299664 | 1.134335 |
| . | suburban - urban | 1.96421 | 0.513428 | Inf | 0.985577 | 3.914582 |

**Table S4.** Model selection table for predictors of pollinator species richness. Models were ranked using AICc, and only models with AIC < 2 are typically considered equally supported. Predictors include garden features, climatic variables, and their interactions

| **Intercept** | **bio1** | **bio12** | **log Garden.size** | **Riq.Pl** | **Type** | **log Garden.size:Type** | **df** | **logLik** | **AICc** | **delta** | **weight** |
| --- | --- | --- | --- | --- | --- | --- | --- | --- | --- | --- | --- |
| 1.846502 |  | 0.000838 |  | 0.028255 |  |  | 4 | -155.876 | 320.8944 | 0 | 0.39793 |
| 1.659571 |  | 0.000874 |  | 0.028435 | + |  | 6 | -154.063 | 322.6716 | 1.777143 | 0.163646 |
| 1.736676 | 0.001234 | 0.000793 |  | 0.028637 |  |  | 5 | -155.691 | 323.1469 | 2.252493 | 0.129028 |
| 1.950162 |  | 0.000877 | -0.02825 | 0.02867 |  |  | 5 | -155.748 | 323.2609 | 2.366489 | 0.121879 |
| 1.377429 |  | 0.000817 | 0.056468 | 0.027276 | + |  | 7 | -153.756 | 325.0112 | 4.116712 | 0.050801 |
| 1.570421 | 0.001035 | 0.000835 |  | 0.028669 | + |  | 7 | -153.926 | 325.3526 | 4.45815 | 0.042828 |
| 1.840716 | 0.001212 | 0.000831 | -0.02762 | 0.029032 |  |  | 6 | -155.57 | 325.686 | 4.791546 | 0.036252 |
| 2.63817 |  |  |  | 0.027399 |  |  | 3 | -160.546 | 327.7582 | 6.863736 | 0.012864 |
| 1.270035 | 0.001105 | 0.000774 | 0.058879 | 0.027473 | + |  | 8 | -153.597 | 327.8387 | 6.944246 | 0.012356 |
| 2.267511 | 0.002963 |  |  | 0.028586 |  |  | 4 | -159.535 | 328.2121 | 7.317651 | 0.010252 |
| 1.166849 |  | 0.000806 | 0.090703 | 0.027331 | + | + | 9 | -152.92 | 329.8406 | 8.946189 | 0.004541 |
| 2.518261 |  |  | 0.024364 | 0.027025 |  |  | 4 | -160.453 | 330.0481 | 9.15364 | 0.004094 |
| 2.516204 |  |  |  | 0.027839 | + |  | 5 | -159.406 | 330.5764 | 9.681981 | 0.003143 |
| 2.187629 | 0.002904 |  | 0.017683 | 0.028299 |  |  | 5 | -159.487 | 330.7383 | 9.843846 | 0.002899 |
| 1.830865 |  |  | 0.114524 | 0.025355 | + |  | 6 | -158.176 | 330.8973 | 10.00287 | 0.002677 |
| 2.155708 | 0.00288 |  |  | 0.0287 | + |  | 6 | -158.421 | 331.3881 | 10.4937 | 0.002095 |
| 1.511177 | 0.002695 |  | 0.112098 | 0.026201 | + |  | 7 | -157.272 | 332.0438 | 11.14938 | 0.001509 |
| 1.136071 | 0.000457 | 0.000789 | 0.0894 | 0.027413 | + | + | 10 | -152.894 | 333.3752 | 12.48074 | 0.000776 |
| 1.407663 |  |  | 0.180544 | 0.025269 | + | + | 8 | -157.229 | 335.1041 | 14.20968 | 0.000327 |
| 1.24125 | 0.002097 |  | 0.165743 | 0.025966 | + | + | 9 | -156.712 | 337.4249 | 16.53045 | 0.000102 |
| 1.356829 |  |  | 0.290181 |  | + |  | 5 | -174.695 | 361.1538 | 40.25937 | 7.2E-10 |
| 1.123239 |  | 0.000335 | 0.277535 |  | + |  | 6 | -174.387 | 363.3203 | 42.42582 | 2.44E-10 |
| 1.269351 | 0.000571 |  | 0.293449 |  | + |  | 6 | -174.676 | 363.8968 | 43.00239 | 1.83E-10 |
| 0.88683 |  |  | 0.363603 |  | + | + | 7 | -174.33 | 366.1593 | 45.26484 | 5.9E-11 |
| 1.13709 | -0.00011 | 0.00034 | 0.276718 |  | + |  | 7 | -174.387 | 366.2735 | 45.3791 | 5.57E-11 |
| 3.291196 |  |  |  |  | + |  | 4 | -178.691 | 366.5249 | 45.6305 | 4.91E-11 |
| 3.579344 |  |  |  |  |  |  | 2 | -181.143 | 366.6094 | 45.71493 | 4.71E-11 |
| 2.695956 |  | 0.000612 |  |  | + |  | 5 | -177.704 | 367.1734 | 46.27897 | 3.55E-11 |
| 3.250093 |  |  | 0.061394 |  |  |  | 3 | -180.912 | 368.4909 | 47.59646 | 1.84E-11 |
| 3.365007 |  | 0.000239 |  |  |  |  | 3 | -180.996 | 368.6588 | 47.76434 | 1.69E-11 |
| 3.743514 | -0.00148 |  |  |  |  |  | 3 | -181.04 | 368.7464 | 47.852 | 1.62E-11 |
| 0.747948 |  | 0.000287 | 0.343268 |  | + | + | 8 | -174.11 | 368.8643 | 47.96984 | 1.53E-11 |
| 3.2863 | 4.06E-05 |  |  |  | + |  | 5 | -178.691 | 369.1466 | 48.25217 | 1.32E-11 |
| 0.895288 | -8.4E-05 |  | 0.363812 |  | + | + | 8 | -174.329 | 369.3037 | 48.40926 | 1.22E-11 |
| 2.80612 | -0.00132 | 0.00066 |  |  | + |  | 6 | -177.621 | 369.787 | 48.89259 | 9.61E-12 |
| 3.424584 | -0.00176 |  | 0.065365 |  |  |  | 4 | -180.768 | 370.6781 | 49.78364 | 6.16E-12 |
| 3.538209 | -0.00228 | 0.00033 |  |  |  |  | 4 | -180.78 | 370.7027 | 49.80826 | 6.08E-12 |
| 3.176195 |  | 0.000147 | 0.050578 |  |  |  | 4 | -180.864 | 370.8717 | 49.97726 | 5.59E-12 |
| 0.803257 | -0.00065 | 0.000309 | 0.343278 |  | + | + | 9 | -174.089 | 372.1785 | 51.28404 | 2.91E-12 |
| 3.355448 | -0.00226 | 0.000238 | 0.048957 |  |  |  | 5 | -180.65 | 373.0647 | 52.17026 | 1.87E-12 |

**Table S5:** Results of the beta models. (A) Beta model with a phylogenetic structure, testing the influence of phylogeny on d.beta. The model includes random effects for species and the phylogenetic component. (B) Beta model testing the effects of species richness, mean annual temperature, annual precipitation, and their interactions on d.beta. The model includes random effects for species and network.

A

| Group-Level Effects: |
| --- |
| ~Phylo (Number of levels: 653) |
| Estimate Est.Error l-95% CI u-95% CI Rhat Bulk_ESS Tail_ESS |
| sd (Intercept) 0.03 0.01 0.00 0.06 1.01 433 1058 |
|  |
| ~SP (Number of levels: 653) |
| Estimate Est.Error l-95% CI u-95% CI Rhat Bulk_ESS Tail_ESS |
| sd (Intercept) 0.90 0.06 0.77 1.03 1.00 1187 2209 |
|  |
| Population-Level Effects: |
| Estimate Est.Error l-95% CI u-95% CI Rhat Bulk_ESS Tail_ESS |
| Intercept -0.23 0.14 -0.48 0.06 1.00 2380 2639 |
|  |
| Family Specific Parameters: |
| Estimate Est.Error l-95% CI u-95% CI Rhat Bulk_ESS Tail_ESS |
| phi 1.84 0.10 1.66 2.04 1.00 4535 5830 |

B

| Group-Level Effects: |
| --- |
| ~NetID (Number of levels: 40) |
| Estimate Est.Error l-95% CI u-95% CI Rhat Bulk_ESS Tail_ESS |
| sd(Intercept) 0.62 0.10 0.44 0.84 1.00 3539 5960 |
|  |
| ~SP (Number of levels: 653) |
| Estimate Est.Error l-95% CI u-95% CI Rhat Bulk_ESS Tail_ESS |
| sd(Intercept) 0.96 0.05 0.86 1.07 1.00 4411 7010 |
|  |
| Population-Level Effects: |
| Estimate Est.Error l-95% CI u-95% CI Rhat Bulk_ESS Tail_ESS |
| Intercept -0.33 0.14 -0.60 -0.06 1.00 3508 5156 |
| log_soma.z -0.10 0.13 -0.35 0.15 1.00 4473 5954 |
| bio1.z 0.06 0.13 -0.20 0.32 1.00 5548 6538 |
| bio12.z 0.08 0.13 -0.19 0.33 1.00 5141 5377 |
| log_soma.z:bio1.z 0.27 0.16 -0.05 0.57 1.00 4779 5823 |
| log_soma.z:bio12.z -0.01 0.16 -0.34 0.30 1.00 4672 6017 |
|  |
| Family Specific Parameters: |
| Estimate Est.Error l-95% CI u-95% CI Rhat Bulk_ESS Tail_ESS |
| phi 2.14 0.12 1.92 2.38 1.00 5864 7269 |
